# The Effect of Yoga on the Quality of Life in the Children and Adolescents with Haemophilia

**Published:** 2015-04

**Authors:** Noushin Beheshtipoor, Shahapar Bagheri, Fatemeh Hashemi, Najaf Zare, Mehran Karimi

**Affiliations:** 1 Department of Pediatrics Nursing, School of Nursing and Midwifery, Shiraz University of Medical Sciences, Shiraz, Iran;; 2 Department of Biostatistics, School of Medicine, Shiraz University of Medical Sciences, Shiraz, Iran;; 3 Haematology Research Centre, Shiraz University of Medical Sciences, Shiraz, Iran

**Keywords:** Quality of life, Haemophilia, Children, Adolescents, Yoga

## Abstract

The problems caused by haemophilia lead to impairments of the quality of life in patients with haemophilia. This study aimed to investigate the effects of yoga on quality of life in the children and adolescents with haemophilia in Shiraz, Iran. This semi-experimental study with pre- and post-test design was performed on 27 boys between 8 and 16 years old who suffered from haemophilia. The patients were divided into two groups. The number of bleedings, absences from school, and referrals to the clinic was evaluated. The demographic data were collected through interviews and using the patients’ records in the haemophilia center. Besides, the quality of life was assessed through the Haemo-QoL questionnaire. Then, the yoga intervention was performed for 14 weeks and the data were collected in three stages. The collected data were entered into the SPSS statistical software, version 18 and were analyzed using non-parametric Friedman test. After the intervention, significant differences were observed in the mean scores of quality of life dimensions and the number of bleedings, school absences, and referrals to the haemophilia clinic (P<0.001). Thus, yoga may improve the haemophilia children’s and adolescents’ perception of quality of life without the risk of injury. This intervention also seemed to be effective in reducing the number of bleedings, referrals to the haemophilia clinic, and absences from school.

## Introduction

Haemophilia is characterized by recurrent bleedings^1^ and its complications lead to impairment of the overall Quality Of Life (QOL).^2^ Individuals with haemophilia are now strongly advised to perform physical activities to improve their physical and mental well-being, particularly in developing countries where factor replacement therapy is limited.^3^ Yoga can play an important role in improvement of QOL in the individuals suffering from chronic diseases.^4^

It should be noted that in Iran, as in most developing countries, access to factor replacement therapy is limited and most patients are treated with on-demand therapy.^5^^,^^6^ Thus, the present study aims to investigate the effect of a 14-week yoga intervention on the life quality of the children and adolescents with haemophilia. The number of bleedings, absences from school, and referrals to the clinic was assessed, as well.

## Materials and Methods

This study was conducted on all the 27 available boys between 8 and 16 years old with haemophilia who had referred to the haemophilia clinic at the children’s hospital in Shiraz, the only haemophilia center in Fars province, southern Iran. Due to the small number of patients and ethical considerations, this study was performed semi-experimentally with pre- and post-test design and the samples were selected through census. The inclusion criteria of the study were having the ability to read and speak Persian, having no prior experiences of yoga, and being able and willing to participate in yoga practice. Written informed consents were obtained from all the children and their parents before the study. This study was approved by the Institutional Human Ethics Committee at Shiraz University of Medical Sciences, Shiraz, Iran. 

The information about the patients’ medical history and demographic characteristics, including the disease and its treatments and number of bleedings, referrals to the haemophilia clinic, and school absences during the last 4 weeks, were collected through a specially-designed questionnaire and the patients’ medical records held at the haemophilia clinic. Moreover, QOL was assessed using the long version of Haemo-QoL questionnaire. The scores of this scale ranged from 0 to 100, with higher values representing lower QOL.^7^

In this study, all the children completed the Persian version of Haemo-QoL. Because the participants in the present study were children and adolescents between 8 and 16 years old, version II or III was used as appropriated. Von Mackensen *et al.* confirmed the reliability of this questionnaire by Cronbach’s alpha of 0.91 and test–retest correlation coefficient of 0.92.^8^ Moreover, Bagheri et al. confirmed that the reliability coefficients for the internal consistency (Cronbach’s alpha) of each of the subscales ranged from 0.61 to 0.81 in children (age group II: 8–12 years old) and from 0.49 to 0.84 in adolescents (age group III: 13–16 years old). Cronbach’s alpha coefficients were also satisfactory for both age groups in the Persian version of the Haemo-QoL questionnaire (0.89 for age group II and 0.78 for age group III).^6^

The intervention was performed for 14 consecutive weeks and consisted of 8 weeks of class attendance (1 h a day, 2 days a week) and 6 weeks of home exercise (1 h a day, 2 days a week). A yoga instructor was trained about the haemophilic children in the haemophilia center. The exercises were selected by consultation with a sports medicine specialist in the haemophilia clinic and** a **specialist in Yoga Sciences. They were also adopted from the yoga videos from the Haemophilia Foundation Australia. The investigator was present in all the yoga classes throughout the 8 weeks in order to monitor the procedure. The yoga classes included warm-up practices, physical postures (asana), a simple practice of controlled breathing (pranayama), and relaxation. After 8 weeks of class attendance, the children were given the yoga video CDs to be able to perform their home exercises for 6 weeks. The participants were reminded and monitored regarding practicing the exercises at home through telephone calls by the yoga instructor and the investigator. None of the children reported any adverse effects regarding the program. The assessments were performed at baseline as well as at the end of the 8^th^ and 14^th^ weeks of the intervention. The data were entered into the SPSS statistical software (version 18) and were analyzed using non-parametric Friedman test. Besides, P<0.05 was considered as statically significant.

## Results

All the 27 boys who met the inclusion criteria were recruited into the study and none was dropped out. Among the study patients, 16 and 11 boys were in the 8-12 and 13-16 years age groups, respectively. Besides, their mean age was 11.5 years (SD=3.9). The descriptive statistics of the study participants have been presented in Table 1.

**Table 1:** Type and severity of haemophilia based on the patients’ age groups

**Haemophilia** ** severity**							
	**Total**	**Age group II** **(8–12 years old)**			**Age group III** **(13–16 years old)**		
		**Mild**	**Moderate**	**Severe**	**Mild**	**Moderate**	**Severe**
Haemophilia A	23	5	2	6	4	2	4
Haemophilia B	4	2	1	-	-	-	1
Total	27	7	3	6	4	2	5

QOL and its dimensions in age groups II and III have been shown in Table 2. Accordingly, the results of Friedman test indicated a significant difference in QOL before the intervention and 8 and 14 weeks after that (X^2^=50.29, P<0.001). Also, the results showed a significant reduction in the mean number of bleeding events (x^2^=44.07, P<0.001), referrals to the haemophilia clinic (x^2^=18.28, P<0.001), and school absences (x^2^=40.13, P<0.001) throughout the assessment stages.

**Table 2:** Comparison of changes in the mean values of Haemo-QoL dimensions in the two age groups and the entire sample before and at the 8th and the 14th week after the yoga intervention (Friedman test)

**Age group II ** **(8–12 years old)** **n=16**					**Age group III** **(13–16 years old)** **n=11**			**All** **n=27**		
**Haemo-QoL**	**Time**	**mean±SD**	** x^2^**	**P**	**mean±SD**	** x^2^**	**P**	**mean±SD**	** x^2^**	**P**
Physical	Pre	59.8±12.5	24.38	<0.001	64.3±10.0	19.46	<0.001	61.6±11.5	43.74	<0.001
	Week 8	47.8±11.5			50.6±8.6			48.9±10.3		
	Week 14	44±9.7			46.4±6.2			45±8.4		
Feeling	Pre	56.9±18.1	26.95	<0.001	66.8±12	16.23	<0.001	60.9±16.4	48.07	<0.001
	Week 8	46.2±17.5			55.7±11.3			50.1±15.7		
	Week 14	43.1±16			49.1±10.3			45.5±14.1		
View	Pre	41.0±13.8	23.61	<0.001	57.0±16.5	21.53	<0.001	47.5±16.7	44.93	<0.001
	Week 8	33.0±11.7			45.9±11.4			38.2±13.1		
	Week 14	30.8±11.3			41.4±10.1			35.1±11.8		
Family	Pre	62.2±12.2	19.50	<0.001	65.6±9.1	15.20	=0.001	63.6±11.0	34.39	<0.001
	Week 8	52.8±9.1			57.7±11.4			54.8±10.2		
	Week 14	50.3±6.7			57.1±11.5			53.1±9.4		
Friends	Pre	72.3±20.2	24.63	<0.001	61.9±9.5	19.15	<0.001	68.1±17.2	43.04	<0.001
	Week 8	57.4±14.1			47.2±8.6			53.2±13.0		
	Week 14	53.9±11.8			46.6±9.8			50.9±11.4		
Perceived support	Pre	61.7±16.1	26.33	<0.001	72.2±14.6	21.14	<0.001	66.0±16.1	47.10	<0.001
	Week 8	52.1±14.3			60.2±10.9			54.9±13.6		
	Week 14	47.3±13.3			53.4±9.0			49.8±11.9		
Others	Pre	32.3±10.9	19.90	<0.001	43.6±12.3	20.18	<0.001	36.9±12.6	38.82	<0.001
	Week 8	25.3±10.7			35.6±11.4			29.5±11.9		
	Week 14	24.5±8.2			31.4±11.1			27.3±9.9		
School and sport	Pre	52.3±13.6	26.83	<0.001	63.1±10.1	22.00	<0.001	57.3±13.0	48.07	<0.001
	Week 8	41.8±10.5			48.7±8.4			44.6±10.1		
	Week 14	39.5±9.5			43.2±7.8			41.0±8.9		
Dealing with haemophilia	Pre	40.2±16.5	25.67	<0.001	33.1±6.0	18.47	<0.001	37.3±13.5	43.92	<0.001
	Week 8	30.1±12.2			21.7±3.4			26.7±10.4		
	Week 14	25.4±11.2			19.8±4.9			23.1±9.5		
Treatment	Pre	50.6±11.7	21.71	<0.001	47.4±9.1	18.47	<0.001	49.3±10.6	40.05	<0.001
	Week 8	39.3±14.5			37.8±11.2			38.7±13.0		
	Week 14	36.8±12.2			34.1±6.9			35.7±10.3		
Future	Pre	-	-	-	48.3±10.9	16.27	<0.001	48.3±10.8	16.27	<0.001
	Week 8	-			38.6±15.3			38.6±15.3		
	Week 14	-			35.2±11.6			35.2±11.6		
Relationship	Pre	-	-	-	44.3±12.9	15.25	<0.001	44.3±12.9	15.25	<0.001
	Week 8	-			34.0±12.6			34.0±12.6		
	Week 14	-			33.0±11.6			32.9±11.5		
Total Haemo-QoL	Pre	53.0±8.7	28.50	<0.001	55.6±4.6	22.00	<0.001	54.1±7.3	50.29	<0.001
	Week 8	42.3±6.1			44.0±4.0			43.0±5.3		
	Week 14	39.5±5.9			40.9±2.7			40.1±4.8		

## Discussion

This study is one of the first researches to support yoga as an intervention to improve the QOL of the children and adolescents with haemophilia. The improvement in QOL demonstrated in our study is consistent with the results of the studies performed on other chronic diseases. For instance, Selvaduria et al*.* showed the positive effects of an exercise intervention on QOL in the children with cystic fibrosis.^9^ Lundgren et al. also suggested that yoga increased QOL in the patients with epilepsy.^4^

In the present study, yoga improved the physical, psychological, and social domains of the haemophilic children’s life. Similarly, in a study by Kuttner et al., children with irritable bowel syndrome exhibited less functional disability, improved acceptance of health, and decreased anxiety following yoga.^10^ Overall, studies have revealed yoga to be effective in improving the physical function, psychosocial impairment, and behavior in both children and adolescents.^11^ Decrease in the frequency of bleeding episodes is noteworthy because it, in itself, has been associated with promotion of QOL in haemophilic patients.^12^ Reduction of bleeding frequency in our study is consistent with the results of the study by Tiktinsky et al.^13^ In that study, this decrease was explained by increase in strength of the muscles around the involved joints. In general, the patients with haemophilia are more vulnerable to stressful conditions which results in creation of new bleeding episodes.^14^ Therefore, increasing resilience to stressful situations can be an important way to reduce the number of bleedings. Parshad^15^ found that yoga helped individuals to become more resilient to stressful conditions. In our study, referrals to the haemophilia clinic and school absences were decreased, too. Brown et al.^12^ found that the frequency of bleeding episodes was negatively associated with QOL. Yoga, thus, may represent a particularly promising approach for improving the lifestyle of the children with haemophilia.

Despite the promising results, our study had some limitations, including the small sample size and lack of a control group. Moreover, the short study period limited the possibility to completely evaluate the maintenance of the improved QOL. Thus, a controlled study is required to be performed on a larger sample size in order to confirm the suggested benefits of yoga in haemophilia patients. Besides, semi-experimental studies including control groups are needed to be conducted in future in order to come to more reliable conclusions. 

## Conclusion

The present study supported the probable effectiveness of yoga not only in increasing the QOL, but also in decreasing the frequency of the bleeding episodes, referrals to the clinic, and absences from school in the haemophilia patients. Although all the study patients were being treated by on-demand therapy, no complications of yoga were reported, indicating that yoga is equally safe for the patients with haemophilia. Thus, the importance of yoga should be taught to the young haemophilics, especially in the countries where there is limited access to clotting factors. 
